# Editorial: Optimal perioperative management of urothelial carcinoma

**DOI:** 10.3389/fonc.2023.1224983

**Published:** 2023-05-31

**Authors:** Ariane Holzman, Elliott Tokarski, Benjamin Pradère, Evanguelos Xylinas

**Affiliations:** ^1^ Department of Urology, Bichat Claude Bernard Hospital, Paris Cité University, Paris, France; ^2^ Department of Urology UROSUD, La Croix du Sud Hospital, Quint-Fonsegrives, France

**Keywords:** urothelial, perioperative, chemotherapy, immunotherapy, adjuvant, neoadjuvant

Urothelial carcinoma (UC) is a prevalent urologic condition arising from the upper (UTUC) to the lower urinary tract (NMIBC and MIBC) that holds for the clinicians several challenges. UC boasts the highest recurrence rate of any malignancy inducing medico-economic challenges towards its surveillance and therapeutic strategies. Recently, the field of UC is undergoing a remarkable transformation due to the development of new therapeutic targets and treatment strategies that holds promise for enhancing the outlook for patients, despite the disease still having a poor prognosis. The objective of this Research Topic was to offer insight into how systemic and local therapies have been integrated into the optimal perioperative management of UC.

Intravesical bacillus Calmette-Guérin (BCG) immunotherapy is currently considered the gold standard adjuvant treatment for high-risk NMIBC. However, BCG-unresponsiveness remains a day-to-day challenge. Current international guidelines consider early radical cystectomy (RC) as the gold standard and only therapeutic approach towards cancer control. However, this procedure comes with high morbidity and mortality rates even in high-volume reference centers. Thus, several novel agents are being tested in ongoing clinical trials for BCG-unresponsive NMIBC. Carcinoma *in situ* (CIS) is high-risk and prone to relapse and progression. Valrubicin (1) was the only approved treatment for BCG-resistant CIS for over 20 years. Several intravesical chemotherapeutic agents have been assessed for BCG failure NMIBC, either alone or in combination (2, 3), but their efficacy profiles have been inconsistent due to the heterogeneous groups of patients included in the trials. To encourage clinical trials, the FDA issued guidelines allowing single-arm studies with complete response rate (CR) and duration of response as key efficacy measures for BCG-unresponsive CIS with or without Ta or T1. In this context, Pembrolizumab, a PD-1 inhibitor previously used as second line therapy for advanced UC, was approved by the FDA to treat BCG-unresponsive high-risk NMIBC, based on KEYNOTE-057 phase II trial (4) results showing a 41% CR rate. Atezolizumab, a PD-L1 inhibitor, is also being studied with promising results (5). Nadofaragene firadenovec (N803), a promising intravesical gene therapy, has shown over 50% complete response with almost half of the patients remaining free from high-grade recurrence at 12 months in a phase III trial of 151 patients (6). This treatment offers the advantage of having an optimal tolerance profile and being effective with a simplified administration schedule. More recent clinical studies in BCG-unresponsive NMIBC patients have mainly focused on the efficacy of the combination of various treatment modalities. For instance, an ongoing phase II multicenter study investigating the use of immune cell– activating interleukin-15 (IL-15) superagonist Nogapendekin alfa inbakicept (NAI) in combination with BCG found a 71% CR rate (7). Also currently, TAR-200 and cetrelimab are being evaluated in a randomized multicenter phase 2 study (8). While the shortage of BCG has also highlighted the urgent need for alternative treatments for high-risk NMIBC, clinicians must weigh the risks and benefits of delaying radical surgery against the potential benefits of novel bladder-sparing therapies.

Non-metastatic MIBC harbors a high risk of disease recurrence and cancer-specific mortality, with around 50% of patients experiencing disease recurrence within 5 years of surgery. Neoadjuvant cisplatin-based chemotherapy (9) may be beneficial, but overall survival benefits remain modest ranging from around 6 to 8% (10). Patients who cannot receive cisplatin-based chemotherapy have limited options. Immune checkpoint inhibitors have been approved for second-line treatment of metastatic UC and for first-line treatment of cisplatin-ineligible patients, providing new hope for previously untreatable patients (11, 12). In the treatment of non-metastatic MIBC, incorporating short courses of pre-radical cystectomy (RC) immunotherapy is a promising new neoadjuvant therapy strategy, especially in patients unfit to cisplatin-based chemotherapy. In two phase II single-arm trials (13, 14), pembrolizumab and atezolizumab were studied as mono-immunotherapies, both reporting significant complete response rates of 42% and 29%, respectively. These rates are comparable to those obtained with neoadjuvant chemotherapy. Furthermore, several prospective, randomized, phase III studies are currently planned to compare chemotherapy with chemoimmunotherapy or combination immunotherapy, such as CTLA4 associated with anti-PDL1, which could establish new standard therapies for the treatment of MIBC. Adjuvant chemotherapy for urothelial cancers is still controversial due to limited and inconclusive data on its efficacy. Adjuvant immunotherapy with immune CPIs is being evaluated in several phase III trials as a potential alternative strategy for patients with residual cancer disease after RC. In the CheckMate 274 trial (15), nivolumab showed a significant improvement in specific survival compared to placebo, especially in patients with PD-L1 expression ≥ 1%. However, the IMvigor010 trial (16), which evaluated atezolizumab in similar conditions, did not meet the primary endpoint of specific survival. Results from the AMBASSADOR trial with pembrolizumab are pending, and overall survival data are anticipated to be limited to patients with PD-L1 expression ≥ 1%. Achieving a complete response (CR) in a neoadjuvant setting is considered a significant endpoint. However, the hypothesis that it may portend a survival benefit remains unproven as the long-term outcomes of patients who achieved CR with neoadjuvant chemotherapy (NAC) or immunotherapy are not well established. Therefore, it is essential to adequately screen patients to determine who would benefit the most from either chemotherapy or immunotherapy in the neoadjuvant setting. This requires a better understanding of the biological mechanisms of response to different treatments and the development of biomarkers to predict response to therapy.

Upper tract urothelial carcinomas (UTUCs) are uncommon and have a poor prognosis. While kidney-sparing surgery is indicated in low-risk UTUC, Radical NephroUreterectomy (RNU) remains the standard of care for high-risk UTUC. More than 50% of patients with progressive high risk UTUC die, despite systemic platinum-based chemotherapy following local or metastatic recurrence. Improved management of early-stage disease, therefore, has the potential to improve patient’s outcomes. Since UTUC shares several clinicopathological features with MIBC and that survival improvements were seen with platinum-based chemotherapy; similar benefits of platinum-based chemotherapy was expected for UTUC. The POUT TRIAL (17), a phase 3, parallel group, open-label, randomized controlled trial, demonstrated improved disease-free survival among patients with muscle-invasive and/or lymph node involvement who received adjuvant cisplatin-based chemotherapy following RNU compared to RNU alone. However, the eligibility to receive an adequate chemotherapy after surgery is a major concern due to the inherent loss of renal function. Despite a risk of overtreatment related to the difficulties of preoperative staging, neoadjuvant platinum-based chemotherapy has the theoretical advantage to be administered to a greater number of patients. A small, single-arm trial (18) demonstrated that neoadjuvant chemotherapy (NAC) was associated with a high rate of downstaging and a low rate of positive surgical margins in patients with high-grade upper tract urothelial carcinoma (UTUC). To confirm the findings and establish a consensus between adjuvant and neoadjuvant cisplatin-based combination chemotherapy (CBCT), larger randomized trials are necessary but difficult to design due to the paucity of the disease.

In conclusion, the optimal perioperative management of urothelial carcinoma requires clinicians to navigate the challenges posed by high-recurrence rates and limited treatment options. New therapeutic targets and treatment strategies such as immune checkpoint inhibitors and intravesical gene and immuno-therapies offer hope for enhancing outcomes for patients with urothelial carcinoma, and ongoing clinical trials evaluating combination therapies may establish new standards of care.

## Author contributions

The four authors made substantial contributions to the conception or design of the work, drafting the work or revising it critically for important intellectual content. All authors provided approval for publication of the content. All authors agree to be accountable for all aspects of the work in ensuring that questions related to the accuracy or integrity of any part of the work are appropriately investigated and resolved.
